# Assessing the Specificity and Accuracy of Accent Judgments by Lay Listeners

**DOI:** 10.1177/00238309221101560

**Published:** 2022-06-19

**Authors:** Natalie Braber, Harriet Smith, David Wright, Alexander Hardy, Jeremy Robson

**Affiliations:** School of Arts and Humanities, Nottingham Trent University, UK; De Montfort University, UK

**Keywords:** Accent recognition, earwitness testimony, accents

## Abstract

Historically, there has been less research carried out on earwitness than eyewitness testimony. However, in some cases, earwitness evidence might play an important role in securing a conviction. This paper focuses on accent which is a central characteristic of voices in a forensic linguistic context. The paper focuses on two experiments (Experiment 1, *n* = 41; Experiment 2, *n* = 57) carried out with participants from a wide range of various locations around the United Kingdom to rate the accuracy and confidence in recognizing accents from voices from England, Scotland, Wales, Northern Ireland, and Ireland as well as looking at specificity of answers given and how this varies for these regions. Our findings show that accuracy is variable and that participants are more likely to be accurate when using vaguer descriptions (such as “Scottish”) than being more specific. Furthermore, although participants lack the meta-linguistic ability to describe the features of accents, they are able to name particular words and pronunciations which helped them make their decision.

## 1 Introduction

Voices contain socially relevant information, and when we listen to a voice, we may make judgments about the speaker, for example, about their occupation, background, personality, and likeability (see also Bent et al., 2016, p. 104). Some aspects of voice are particularly important in terms of providing clues about a person’s identity which can be especially relevant in a forensic situation. Witnesses to a crime may be asked to describe the perpetrator, and in cases where the witness hears but never sees this person, they may be asked by the police to provide information about identifying features of the voice they heard. Accent is frequently reported by witnesses (Kerstholt et al., 2006, p. 189) and a lay listener’s^1^ perception of accent can be crucial to a police investigation and may subsequently form part of the evidence against a suspect in a prosecution. The outcome of the prosecution may in part be determined by the jury’s assessment of how accurate that perception was. In England and Wales, juries are directed in cases concerning voice identification to consider whether there were any factors which made a suspect’s speech distinctive (Judicial College, 2020, Section 15.30). Accent is likely to play a part here. A witness’ ability to identify an accent may be an important issue in a criminal trial. In Nealon [2014 EWCA 574], the defendant, who had a distinctive Irish accent, was convicted of rape despite the fact that a number of witnesses described the attacker as having a Scottish accent. Nealon was exonerated on the basis of DNA evidence after serving 16 years in prison. There is currently no standard protocol for obtaining voice descriptions from earwitnesses, although there are some guidelines (see Nolan & Grabe, 1996 for more information). In this paper, we examine the specificity and accuracy of lay listeners in detecting different regional accents and how confident they are in their recognition. We are interested to examine which linguistic features they use to come to their decisions.

Generally, eyewitness testimony has been the focus of much research and there is less research on earwitness testimony (see, for example, Broeders, 1996; Bull & Clifford, 1999; Clifford, 1980). Cases relying on earwitness testimony are relatively rare compared to eyewitness testimony (Bull & Clifford, 1999, p. 195). Nevertheless, when we listen to voices we decode a wide range of social information, and our judgment can be affected by different factors (such as our experience of different voices) which could be of crucial importance in legal contexts. We start by examining relevant studies which consider the importance of accent and accent recognition in the field before outlining our two experiments and their findings.

## 2 Literature review

### 2.1 Accents

Hanani et al. (2013, p. 59) comment that “[a] speech signal contains a wealth of information over and above its linguistic content, including clues to the geographical, social and ethnic background of the speaker.” Specifically, regional accents, which affect a speaker’s pronunciation, make up an important part of a speaker’s social identity (Beal, 2006, p. 15; Bestelmeyer, 2019, p. 667; Gluszek & Dovidio, 2010a, p. 215). It is important to state that all speakers have accents (Derwing & Munro, 2008, p. 476) and that there is nothing inherent (linguistically) in particular accents which make them more pleasing than others (Gluszek & Dovidio, 2010a, p. 217).

Listeners are aware of regional accents and we know this as they are named by non-linguists when asked to describe other speakers’ voices (Bent et al., 2016, p. 104). People describe accents as being “northern” or “Irish,” for example, but that does not mean that there is only one variety used in those regions. It does mean, though, that speakers in such a region have enough features in common which are not shared with other varieties. We can find more local labels, such as, “Yorkshire,” “Glaswegian,” or “Brummie,” which suggest that smaller areas still show enough differences to be noticeable to listeners. That is not to say that we can draw a line between different varieties but that there are gradual patterns of variation (Hughes et al., 2005, p. 9).

Previous research on the social impact of accents has mainly focused on bias and how speakers use accent to make evaluations about speakers (see Gluszek & Dovidio, 2010b, p. 224). Many years of research from the 1970s onwards (see, for example, Giles, 1970) has shown that listeners use stereotypes to attach attributes to particular accents and judge their speakers (Bishop et al., 2005, p. 131). There are values and stereotypes associated with particular accents as listeners make judgments based on preconceived ideas (Coupland & Bishop, 2007, p. 74; Dixon et al., 2002, p. 162; Edwards, 1982, p. 20) which can be positive or negative. Furthermore, it seems that “specific linguistic variants are associated in the minds of speakers and hearers with particular social characteristics” (Beal, 2010, p. 87). Most social psychological research into accents has focused on how listeners perceive speakers based on characteristics of particular accents (Gluszek & Dovidio, 2010a, p. 217). For example, whereas standard varieties are associated with intelligence, some industrial urban varieties have negative associations regarding education and employment and rural voices are judged to be likable but unintelligent (Beal, 2006, p. 29). For a full discussion of such research, see Dragojevic et al. (2021) who give a detailed overview of the last century.

### 2.2 Recognizing accents

Accent is a very important aspect of recognizing voices, both for humans and computers (see Arslan & Hansen, 1996, p. 355) although humans have been shown to perform at a lower level than automatic systems (Hanani et al., 2013, p. 70). The fact that computers can recognize accents with a small amount of data suggests that the differences are significant enough to be detected automatically by computers (Hanani et al., 2013, p. 73). From a young age, humans tune into language: infants under 6 months can discriminate phonemic contrasts which are not present in their “ambient” language (Clopper & Pisoni, 2004a, p. 31), and infants as young as 5 months show preference for their own accent over others (Gluszek & Dovidio, 2010a, p. 215). Other studies show that young infants can distinguish between their home accent and another accent, but not between two unfamiliar regional accents (Butler et al., 2011, p. 392). Research has also shown that hearing one’s own accent (not only our own voice) is associated with increased neural activation, suggesting a stronger emotional reaction to speakers with similar accents to our own (Bestelmeyer et al., 2015, p. 3956).

Further studies suggest that people are better at recognizing familiar accents (Arslan & Hansen, 1996, p. 365; Braun et al., 2018, p. 248; Doty, 1998, p. 202; Hanani et al., 2013, p. 70; Smith, 2013) and listeners are better at recognizing native speakers than speakers using a non-native language (Doty, 1998, p. 204), although there is some contradictory evidence (see, e.g., McKenzie et al., 2019) which suggests this could be due to levels of previous exposure. Ikeno and Hansen (2006, p. 401) explain that listeners’ accent background impacts on perception, as well as comprehension of what is said. By default, therefore, less familiar accents are detected at a lower level of accuracy (Ikeno & Hansen, 2006, p. 402). Shen and Watt (2015, p. 107) add that non-native speakers are better at recognizing and discriminating the origins of other non-native speakers speaking English than English L1 speakers are. McKenzie (2015) also found that native speakers of English were better at recognizing where other L1 speakers were from and found it harder to identify where non-native speakers came from. Non-native accents are often rated more negatively and threateningly, thus making them more vulnerable to negative perceptions (Birney et al., 2020, p. 495; Roessel et al., 2020, p. 87). It seems listeners expect non-native accents to be more difficult to process and judge negatively when asked about comprehensibility and fluency (Dragojevic & Giles, 2016, p. 415).

In terms of recognition and discrimination of accents there are also other issues. Speakers of regional accents are more familiar with the standard than vice versa (Adank et al., 2009, p. 521; Braun et al., 2018, p. 233; Griffiths, 2010) and it seems that certain types of accents are more confusable than others (Ikeno & Hansen, 2006, p. 403). Varieties closer to the standard are very hard to place regionally (Hanani et al., 2013, p. 72) and listeners tend to be better at defining dialect regions closer to them as opposed to those further away (Clopper & Pisoni, 2004b, p. 113; Dragojevic, 2018, p. 7). This means that some accents may be recognized only at a more general level (e.g., Scottish), whereas, others may be defined much more narrowly and this will vary between people. Most perceptual research in linguistics does not consider geographical knowledge, and many such studies ask participants to make judgments about the speakers or their language usage without asking them to listen to speech (Clopper & Pisoni, 2004b, p. 114). It was frequently assumed that people could recognize accents with reasonable accuracy, so that, such recognition was not often checked (Garrett et al., 2003, p. 198; see also Dragojevic et al., 2018).

People are good at detecting an imitated accent (as opposed to someone’s own accent) as it seems people have prototypes of accents and there is much similarity across a speaker group (Neuhauser & Simpson, 2007). People may use stored exemplars and map speakers onto these to aid with recognition (Evans & Iverson, 2004, p. 352). Adults are more accurate than young people, which is likely to be linked to more experience of different accents added to geographical mobility (Clopper & Pisoni, 2006; Garrett et al., 2003, p. 201).

Although research shows that listeners may be able to accurately make very broad dialect clusters (such as accents being American or British English), they find it much harder to categorize into smaller regions (Clopper & Pisoni, 2004b, p. 111) and some accents are easily confused, for example, Scottish and Irish (Tompkinson & Watt, 2018, p. 31) which could also be related to exposure to such varieties. Accuracy in such recognition tasks can be very low and Clopper and Pisoni (2004b, p. 116) question whether listeners have knowledge of other varieties’ phonology. However, Watt (2010, p. 78) suggests that listeners are very good at internalizing different voices, so that, unfamiliar voices and accents can become quickly familiar. Contact through the media can also help recognition of accents that are not local (Stevenage et al., 2012, p. 649).

Clopper and Pisoni (2004a) question whether where a person lives affects accuracy (see also Clopper and Pison 2004b, p. 137; Clopper & Pisoni, 2007; Doty, 1998, p. 206). However, recognition could go beyond solely residency and be affected by our wider experiences of different varieties of language due to life experience (Bent & Holt, 2017, p. 2; Sumner & Samuel, 2009, p. 498). There is also research which suggests that language can evoke strong in-group preferences with individuals viewing others who have similar accents to their own more favorably (Gill, 1994, p. 349). “Other” accents, and particularly non-native ones, are highly salient and a potential cause of stigmatization for speakers (Gluszek & Dovidio, 2010a, p. 214). Creating “in” and “out” groups is an important aspect of social categorization of which accent is just one part (Bestelmeyer et al., 2015, p. 3953).

What we can see from this is that many studies on accent and perception of accent by non-linguists focus on the speakers of these varieties and what these varieties mean socially and psychologically (see also Ladegaard, 2001, p. 25). They focus on the ability to recognize different varieties and the attitudes held toward these (see also Garrett, 2010 as well as Sharma et al., in press) is also seen as crucial. These studies also show us that accent is an important factor of voices. Many of these studies focus on accent recognition and identification but there are also many studies which focus on evaluation and attitudes toward accents (see also Dragojevic, 2018).

This paper will focus on the issue of accuracy in recognition but from a different angle which is not part of these former studies. We will be examining the accuracy of recognition but bringing this together with specificity—what kinds of accent labels do participants use to label voices? Does this vary depending on where the speaker is from? From a forensic angle, there are also linguistic studies which investigate accent evaluation and the evidence this may have on the perception of guilt (see also Dragojevic et al., 2021). Dixon and Mahoney (2004) examined whether nonstandard accents would be perceived as more guilty than standard speakers. Their study included almost 200 undergraduate students listening to four created police interviews, where the “suspect” used a received pronunciation (RP) or Birmingham accent during the interviews. Results showed that more than 95% of their participants were able to guess correctly the suspects’ regional identity (2004, p. 67). This is very high, but more detail is needed to establish what their participants were asked to do. On the other hand, other studies suggest that participants are not very good at recognizing accents (Hollien, 1996, p. 15). This could be due to the fact that people do not have the meta-linguistic knowledge to describe voices, which Griffiths illustrated in an accent description study in a forensic context (Griffiths, 2010, p. 2). Tompkinson and Watt (2018: 19–21) questioned whether people have the ability and language to describe voices. People are generally not aware that they may need to recognize a voice again which is similar to an earwitness context, so that, if witnesses are unprepared or have only little time to encode efficient strategies, then that will be problematic for earwitness testimony (Clifford, 1980, p. 383). The issue of confidence when placing, labeling and describing accents is also important as this is rarely examined within the research but can be used within the court to strengthen or undermine evidence. For example, the defense might argue that an unconfident witness may be mistaken, while the prosecution might argue that a confident witness is likely to be correct. This focus on confidence and its relation to the particular levels of recognition also differentiate this study from previous research. Furthermore, we focus also on the background of the speaker to examine whether where they come from influences the levels of specificity and confidence of their responses.

This paper will focus solely on one particular aspect of the voice, namely regional accent, and listeners’ ability to recognize accents as they may be asked to do as part of their earwitness testimony through two different experiments. We know that accent is a very salient feature of language and would like to know more about how listeners respond to accents they hear. In the two experiments that follow and will each be discussed in turn, we will examine accuracy in accent detection, confidence in reporting accuracy, and identifying which features listeners comment on when making such detections. Our aim is to follow-up on what is already known about accent recognition and try to understand how this type of task can be applied in the real world, for example, when earwitnesses are asked about voices they may have heard to try to find the best way of gathering relevant and accurate evidence. Our research questions are as follows:

- Can speakers detect British English accents accurately?
- Can we find out which linguistic features they use to do so?
- Does speaker confidence reflect accuracy?
- Does the listener’s residence or their experience of an accent affect their identification?

## 3 Experiment 1

In Experiment 1, we asked listeners to make accent detection judgments. We were interested in how accurate and specific their answers were, and how confident they were in their responses. Based on the existing literature, we expected accuracy, specificity, and confidence to vary between different accents depending on the exposure they had with accents from around the United Kingdom, depending on where they were from, where they had lived and experience with such accents in the media.

### 3.1 Participants

There were 41 participants (25 females and 16 males) with an average age of 31.26 years (*SD* = 10.67; age range = 18–61). All participants had normal or corrected-to-normal hearing, were native English speakers and were born and/or raised in the United Kingdom. The participants were recruited using convenience sampling and social media platforms and were invited to enter a prize draw to win Amazon vouchers (£50/£25/£10). Ethical approval was provided by Nottingham Trent University’s (NTU) ethics committee.

### 3.2 Materials

The stimuli were drawn from the Centre for Speech-Technology Research Voice Cloning Toolkit (VCTK) corpus (Veaux & Yamagishi, 2017). This corpus features voice data uttered by 109 native speakers of English with various accents. The 96 kHz versions of the recordings are publicly available at https://doi.org/10.7488/ds/2101. Each speaker reads aloud a selection of about 400 sentences. These include the Rainbow Passage, sentences selected from a newspaper, and an elicitation paragraph intended to reveal the speaker’s accent. Using read speech, rather than a spontaneous speech sample, allowed us to control the content across all speakers, ensuring we isolated accent from other linguistic features, such as morpho-syntax and lexis, which were not the focus of this study (see Van Bezooijen & Gooskens, 1999, which involves the examination of the extent to which prosodic and pronunciation cues are crucial in identifying speakers).

For this experiment, 12 speakers (see Table 1) were selected. We used a single voice to represent each accent because the VCTK corpus does not feature enough speakers from the same specific locations to facilitate stimulus sampling. Selecting speakers from different geographical areas to represent broad accent categories would have introduced noise to the data. Male and female voices were used and all speakers were aged between 18 and 30. They each read an excerpt from the Rainbow Passage with a duration of approximately 10 seconds. The same excerpt was used for each speaker: “If the red of the second bow falls upon the green of the first, the result is to give a bow with an abnormally wide yellow band, since red and green light when mixed form yellow.” This passage is frequently used in linguistic studies as it includes a sufficient range of vowel and consonant sounds to allow comparison between different varieties of English (see, for example, Boyd et al., 2015). Although accents can vary to a great extent even within regions, it may be useful to consider some of the most salient features of the linguistic varieties which participants listened to in Experiments 1 and 2 (for more information, see Wells, 1986) as these are likely to be features focused on by our participants and this will be further examined in the qualitative analysis of Experiment 2, where we ask which words our participants used to place where the voice was thought to be from. A very brief description of these features can be seen in Table 2, although of course not all of these features will apply to all speakers. Both reading passages used in Experiments 1 and 2 contain examples of words which include these vocalic and consonantal features.

**Table 1.** Voices Used From the VCTK Corpus.

Location	Gender
Northern Ireland (Belfast)	Female
Republic of Ireland (Tipperary)	Male
Scotland (Orkney)	Male
Wales (Cardiff)	Female
North-West (Manchester)	Female
North-East (Newcastle)	Male
Yorkshire	Male
East Midlands (Leicester)	Male
West Midlands (Stafford)	Female
East Anglia (Essex)	Male
South-East (Surrey)	Male
RP (Oxford)	Female

**Table 2.** Some Salient Features of Accents Used During These Experiments.

**Scotland** Rhotic; foot/strut split; no h-dropping; t-glottalling; lack of opposition between foot and goose; possible lack of distinction between trap/palm and lot/thought; Scottish vowel length rule	**Wales** Mainly non-rhotic; clear /l/; strut/schwa merger; long face and goat vowels; distinctive nurse vowel; frequently long duration of consonants; musical prosody	**Northern Ireland** Rhotic; no h-dropping; clear /l/; foot/strut split; both /ɵ/ and /ð/ appear as fricatives; distinctive mouth vowel; rising intonation at end of sentences
**Republic of Ireland** Rhotic; clear /l/; no h-dropping; foot/strut split; some pin/pen merger; some price/choice merger; /ð/ and /ɵ/ produced as dentals rather than fricatives	**North-East** Lack of foot/strut split; glottalization of /p,t,k/; no h-dropping; face and goat produced as monophthongs; clear /l/; nurse/north merger for some	**North-West** Lack of foot/strut split; velar nasal plus; nurse/square merger and lack of closure in voiceless stops in Liverpool; generally non-rhotic (except some areas in Lancashire); h-dropping; lack of bath/trap split; no happy tensing.
**East Midlands** Non-rhotic; frequent h-dropping; frequent yod-dropping; lack of bath/trap; split; lack of split in foot/strut; happy as /ɛ/; distinctive letter vowel	**West Midlands** Non-rhotic; velar nasal plus; “fudged” foot/strut split; variable bath/trap split; distinctive price vowel; h-dropping	**East Anglia** Non-rhotic; foot/strut split; bath/trap split; some goose/goat mergers; near/square mergers, t-glottalling; categorial yod-dropping for some
**South-East** Non-rhotic; bath/trap split; foot/strut split; t-glottalling; h-dropping; happy tensing	**South-West** Rhotic; variable bath/trap split; foot/strut split; little t-glottalling; distinctive lot and goat vowels	**Received Pronunciation (RP)** Non-rhotic; bath/trap split; foot/strut split; little t-glottalling; no h-dropping; happy tensing

### 3.3 Procedure

Participants were invited to take part in an experiment investigating how well people can identify accents. Participants for the study were recruited on social media—random sampling from around the United Kingdom, not tied to specific geographical regions as we wanted to ensure a spread of participants from around the country. We ensure that by asking for native speakers or those raised in the United Kingdom, as we know that non-native speakers who have been living in the United Kingdom for a shorter amount of time find accent recognition much more difficult (McKenzie, 2015). We also asked participants about where they lived and whether they had moved around frequently. The average amount of unique locations within Britain and Ireland that participants had reported living in was 2.22 (±1.50 *SD*). The 41 participants that took part had reported living in a total of 131 unique British and Irish locations with a range of either having in lived in just one unique location to seven unique locations. Some participants had also lived in other countries but these were not included.

The experiment ran using Qualtrics and took approximately 15 minutes to complete. Participants were asked to complete the experiment using headphones and were given a code word to type in to evidence that they could hear clearly what was coming out of the headphones. They were also asked not to change the volume of the recordings once this test had taken place.

The participants listened to each of the 12 voices in turn, presented in a random order. They heard each voice once and only those participants whose timings indicated this were included in these data. They were asked where they thought the speaker came from and to provide a response by writing a location or naming the accent and being as specific as they could (for a discussion on the benefits of such a free choice task, see Bent et al., 2016, p. 105). Following this, they were asked how confident they were in their judgment (on an 11-point rating scale: 0 = *not confident at all*; 11 = *extremely confident*). They were not able to proceed to the next speaker until they had listened to the entire extract and completed the questions.

### 3.4 Results and analysis

As we were interested in examining how specific the responses were that participants gave, we coded the responses given by the participants according to the following levels:

1. Country (England, Scotland, Wales, Ireland)
2. Large area (e.g., South England, Northern Ireland)
3. Region (e.g., East Midlands, North West England, East Anglia)
4. County (e.g., Yorkshire, Leicestershire)
5. City (e.g., Glasgow, London, Manchester)

The percentage of responses provided at each level of specificity is presented in Table 3. Please note, the percentages in Columns 1–5 do not indicate accuracy, but rather how specific participants were when providing a response. Overall accuracy for each accent is provided in the “Accuracy (%)” column. For example, Table 3 indicates that for the Cardiff accent, 36.59% of the responses were provided at Level 5 (city), but only 14.63% of the overall responses were accurate. In Table 3, we present overall accuracy, but it is worth noting that only 9.62% of responses provided at Level 5 (city) were correct, while 80.64% of responses provided at Level 1 (country) were correct. Even random performance (i.e., guessing) at Level 1 would lead to 25% accuracy given there are only four options. This is not true of Level 5, as there are far more possible options. Despite this, we treat all incorrect responses in the same way as any incorrect response has the potential to be misleading in terms of identity. In a forensic context, an incorrect response from a witness could derail an investigation, regardless of the level of region specificity.

**Table 3.** Listener Responses: Level of Region Specificity, Accuracy, and Confidence.

Accent	Level of region specificity (%)					Accuracy (%)	Confidence (%)
	1	2	3	4	5		
Belfast	42.50	40.00	2.50	2.50	12.50	64.10	58.97
Tipperary	60.53	23.68	7.89	0.00	7.89	71.05	67.22
Orkney	64.10	0.00	5.13	0.00	30.77	61.54	71.84
Cardiff	19.51	19.51	12.20	12.20	36.59	14.63	40.00
Manchester	7.89	31.58	7.89	18.42	34.21	13.16	39.43
Newcastle	22.22	22.22	8.33	16.67	30.56	25.00	40.31
Yorkshire	0.00	18.42	5.26	23.68	52.63	35.29	46.05
Leicester	2.50	30.00	17.50	0.00	50.00	15.38	44.50
Stafford	7.89	31.58	13.16	7.89	39.47	22.86	44.12
Essex	2.70	18.92	8.11	18.92	51.35	24.32	44.44
Surrey	5.00	32.50	12.50	12.50	37.50	55.00	47.30
Oxford	4.88	31.71	12.20	14.63	36.59	39.02	47.11

**Figure 1.**
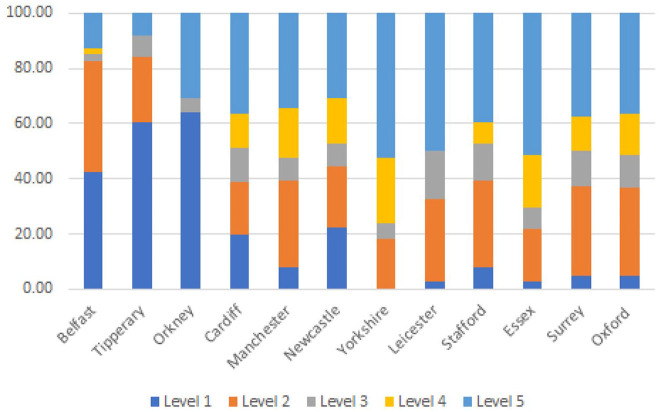
Levels of region specificity given for each accent: Experiment 1.

Accuracy was recorded at the level given by the participants. Therefore, if a participant responded with “Scotland” for the Orkney voice, this would be marked as correct, but incorrect if they had answered with “Edinburgh.” The descriptive statistics reported in Table 3 reveal that overall accuracy was low, but that it varied across accents. Overall, participants were not particularly confident in their responses. The least accurate responses were for Manchester (13%), Leicester (15%), and Cardiff (15%). The highest accuracy was for Tipperary (71%), Belfast (64%), and Orkney (62%). It appears that less specific responses were more likely to be accurate, so that, for the three most accurate voices, the overwhelming responses were at Level 1 where respondents were saying that the voices were “Irish” or “Scottish,” but very rarely were more detailed responses given. For the voices from England, participants were more likely to give a more detailed response (such as Liverpool, Manchester, or London), but accuracy was low (see also Bent & Holt, 2017 for a discussion about broader and more specific distinctions related to accuracy). The percentage of responses for each level can be more clearly visualized in Figure 1.

As the responses are likely to vary across both speakers and listeners, we explored the accuracy data using multilevel modeling, so that, both listeners and speakers could be treated as random effects (see Smith et al., 2019). The relationship between level and accuracy was examined using the ordinal package in R (Christensen, 2011). Level was the dependent variable and accuracy was the predictor. Two models were compared, the first only included intercepts, and the second included accuracy as a predictor. Level predicted accuracy (*G*^2^ = 102.49, *p* < .001). In line with what we expected based on the visual inspection of Table 1, less specific responses were more likely to be accurate.

We examined the relationship between confidence and accuracy using the same method that we used to examine the relationship between level and accuracy. Confidence predicted accuracy (*G*^2^ = 26.46, *p* < .001); responses provided with higher levels of confidence were more likely to be accurate.

All R-scripts and data can be accessed at: https://osf.io/ezmr3/?view_only=321a4d22b82745fda0fa54b617a99267.

In summary, accuracy was low, but variable. This reflects that accent identification can be difficult, but that some voices are easier to classify than others. The difficulty of accent identification is also reflected by the low confidence ratings. It appears that listeners have metacognitive awareness of the difficulty of accent identification; there is a relationship between confidence and accuracy. Responses varied in terms of how specific they were and were more likely to be accurate if they were less specific. This may be because people have a tendency to be vague when they are not sure or are not particularly familiar with the accent in question. It is notable that the non-English accents elicited some of the least specific (and therefore most accurate) responses. It would appear that erring toward being vague can be a sensible strategy in terms of achieving accuracy which might be of particular importance in a forensic setting.

## 4 Experiment 2

For our purpose, the VCTK has some limitations. For example, it does not provide precise location information for all voices (e.g., only “Yorkshire” for one of the voices), making it difficult to examine listener’s performance in terms of how accurate or specific they were. Furthermore, the speakers do not all have particularly “strong” local accents determined by distinctive vowel and consonant pronunciations. As overall accuracy was low in Experiment 1, we decided to record speakers with more distinctive regional accents for Experiment 2, and to use longer speech samples. We also examined the relationship between speaker and listener location, recruiting both English and Scottish participants, and examining how they responded to English and non-English speakers. Based on the results of Experiment 1, we expected that listeners would respond to the accents they were least familiar with by being less specific. We examined which linguistic features and specific words listeners used to inform their responses by asking them how they had come to a decision regarding speaker accent. We also asked questions about their familiarity with these accents and where they had heard them before.

### 4.1 Participants

There were 57 participants (36 females and 21 males). The average age was 35.89 years (*SD* = 12.64; age range = 19–66). Of these 35 were born and/or raised in England, and 22 were born and/or raised in Scotland. As in Experiment 1, all participants had normal or corrected-to-normal hearing, and were native English speakers. We requested native speakers or those raised in the United Kingdom, as we know that non-native speakers who have been living in the United Kingdom for a shorter amount of time find accent recognition much more difficult (McKenzie, 2015). We also enquired about the extent to which participants had moved around the United Kingdom. The average amount of unique locations within Britain and Ireland that participants had reported living in was 2.71 (+–1.46 *SD*). The 57 participants that took part had reported living in a total of 152 unique British and Irish locations with a range of either having in lived in just one unique location to six unique locations. Some participants had also lived in other countries, but these were not included. The participants were recruited using convenience sampling and social media platforms and were invited to enter a prize draw to win Amazon vouchers (£50/£25/£10). Ethical approval was provided by NTU’s ethics committee.

### 4.2 Materials

The voice samples featured speakers who were staff at NTU. All the speakers were male, aged between 35 and 55, and all had regional accents which could be seen as typical of the region they represented. One of the limitations of the first experiment was that both male and female voices were rated (see also other studies such as Carrie, 2017 where gender is included to test for evaluative differences). Due to the possible confound of accent with the gender of the speaker in Experiment 1 (as has been suggested by Garrett, 2010, p. 94), we ensured gender was controlled in Experiment 2. The regional background of these 12 speakers can be seen in Table 4. In a similar way to Experiment 1, we use one speaker to represent one accent. All voice samples were sampled at accent level, which reduces the ability to sample speakers as adding significantly more speakers would have meant having to reduce the range of voices to ensure the experiment could be completed within a realistic timeframe. We ensured the voices used were agreed by all co-authors as being representative of that region—the team has much experience in working with accents and all have a range of accents from around the United Kingdom.

**Table 4.** Voices Recorded for the Project.

Location
Northern Ireland (Belfast)
Republic of Ireland (County Kerry)
Scotland (Giffnock, near Glasgow)
Wales (Carmarthen)
North-West (Bolton)
North-West (Liverpool)
North-East (Durham)
East Midlands (Nottingham)
West Midlands (Birmingham)
South-West (Bristol)
South-East (North London)
RP (Welwyn Garden City)

The speakers were recorded reading aloud an extract from the Rainbow Passage. The extract had a duration of approximately 30 seconds:

When the sunlight strikes raindrops in the air, they act as a prism and form a rainbow. The rainbow is a division of white light into many beautiful colours. These take the shape of a long round arch, with its path high above, and its two ends apparently beyond the horizon. There is, according to legend, a boiling pot of gold at one end. People look, but no one ever finds it. When a man looks for something beyond his reach, his friends say he is looking for the pot of gold at the end of the rainbow.

### 4.3 Procedure

Participants were invited to take part in an experiment investigating how well people can identify accents. The experiment ran using Qualtrics and took approximately 20 minutes to complete. Participants were asked to complete the experiment using headphones and were given a code word to type in to evidence that they could hear clearly what was coming out of the headphones. They were also asked not to change the volume of the recordings once this test had taken place.

After these instructions, the participants listened to each of the 12 voices in turn, presented in a random order. The written extract of text was visible while each voice was playing. The participants were asked where they thought the speaker came from, and to be as accurate and specific as possible. Following this, they were asked how confident they were in their judgment (on an 11-point rating scale: 0 = not confident at all; 11 = extremely confident). They were also asked:

- to write down any words or phrases that helped them identify where the speakers came from
- how the way the person spoke informed their decision (e.g., particular voice features)
- how they knew what the accent was (options: they had heard this accent before on radio/TV/media; they knew someone with the same accent; they had lived in location with the same accent; other which could then be expanded on)
- whether the voice itself was familiar to them, that is, whether they knew any of the voices personally (this was not the case for any of the participants).

The participants were not able to proceed to the next speaker until they had listened to the entire extract and completed all of the questions.

### 4.4 Results

#### 4.4.1 Quantitative analysis

As in Experiment 1, responses were classified according to level of regional specificity, as well as accuracy. The percentage of responses provided at each level of specificity is presented in Table 5. The percentages in Columns 1–5 do not indicate accuracy, but rather how specific participants were when providing a response. Overall accuracy for each accent is provided in the “Accuracy (%)” column. For example, Table 5 indicates that for the Durham accent, 76.19% of the Scottish listeners’ responses were provided at Level 5 (city), but only 14.29% of the overall responses were accurate (meaning that they named another location in the region, for example, Newcastle).

**Table 5.** Accuracy and Confidence Levels.

Accent	Listener	Level of region specificity (%)					Accuracy (%)	Confidence (%)
		1	2	3	4	5		
Belfast	Scot	4.55	40.91	4.55	0.00	50.00	81.82	80.95
	Eng	31.43	42.86	5.71	0.00	20.00	88.57	76.25
Birmingham	Scot	0.00	22.73	9.09	9.09	59.09	50.00	51.36
	Eng	0.00	14.71	8.82	11.76	64.71	44.12	50.34
Bolton	Scot	0.00	4.55	0.00	72.73	22.73	18.18	60.95
	Eng	0.00	0.00	0.00	70.59	29.41	11.76	62.67
Bristol	Scot	4.76	33.33	14.29	14.29	33.33	23.81	34.74
	Eng	6.25	18.75	18.75	15.63	40.63	37.50	41.60
Carmarthen	Scot	45.45	22.73	9.09	0.00	22.73	59.09	80.50
	Eng	55.88	20.59	0.00	0.00	23.53	73.53	78.33
Co Kerry	Scot	18.18	45.45	0.00	4.55	31.82	36.36	70.48
	Eng	42.86	28.57	5.71	2.86	20.00	54.29	69.06
Durham	Scot	0.00	0.00	4.76	19.05	76.19	14.29	69.05
	Eng	3.03	3.03	15.15	12.12	66.67	24.24	61.56
Glasgow	Scot	0.00	4.55	4.55	4.55	86.36	22.73	69.50
	Eng	54.55	3.03	0.00	0.00	42.42	63.64	67.81
Liverpool	Scot	0.00	0.00	0.00	9.52	90.48	90.48	75.00
	Eng	0.00	0.00	5.71	17.14	77.14	60.00	60.91
N London	Scot	13.64	27.27	13.64	18.18	27.27	63.64	54.21
	Eng	2.94	14.71	32.35	20.59	29.41	55.88	47.33
Nottingham	Scot	0.00	14.29	9.52	0.00	76.19	19.05	45.00
	Eng	0.00	9.38	12.50	21.88	56.25	31.25	41.82
W G City	Scot	4.55	22.73	4.55	18.18	50.00	31.82	45.50
	Eng	0.00	14.71	29.41	20.59	35.29	47.06	49.33

The descriptive statistics presented in Table 5 reveal that both specificity and accuracy were highly variable, with many voices eliciting different patterns of responses for English and Scottish listeners. Confidence was also highly variable.

Table 5 illustrates some of the differences between Scottish and English participants. For example, where just over 50% of English participants used the category “Scottish” for the Glaswegian voice, no Scottish participants used such a vague descriptor and almost all participants used Level 5 to describe the voice. We see similar patterns for the two Irish speakers where the Scottish participants use the descriptor “Irish” at 4% and 18%, whereas the English participants are more likely to use Level 1, with 31% and 43%, respectively for the Irish varieties. The exception to the non-English voices is the Carmarthen voice, where both English and Scottish participants use Level 1 (Wales) at around 50%. We can also see that for the English voices, the category “English” is almost never used by the participants, neither Scottish nor English. Confidence levels are similar across Scottish and English participants.

**Figure 2.**
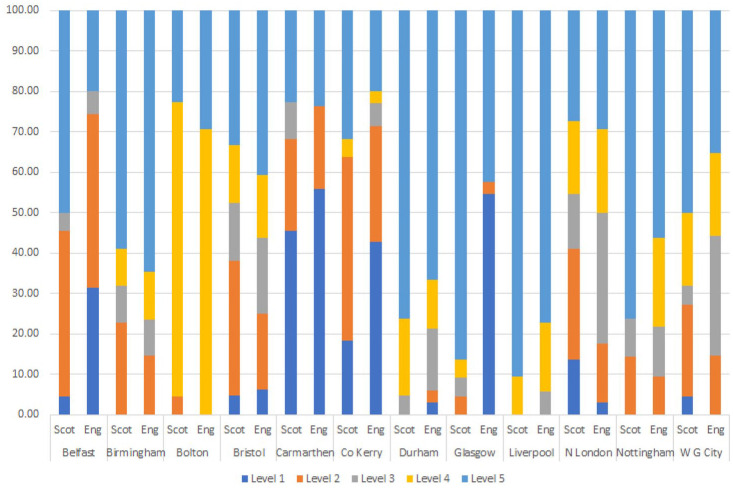
Level of region specificity given for each accent: Experiment 2.

We can see the levels allocated by the Scottish and English participants for each individual voice in Figure 2.

Accent identification accuracy was analyzed using multilevel logistic regression (lme4 package in R: Bates et al., 2015). Both listeners and speakers (i.e., accents) were treated as random effects. We compared three nested models, fitted using restricted maximum likelihood. Accuracy (0 or 1) was the dependent variable. Model 1 included a single intercept, Model 2 included the main effects (listener nationality and speaker nationality), and Model 3 included the two-way interaction. The likelihood ratio tests, provided by lme4, were obtained by dropping each effect in turn from the appropriate model. The chi-square statistic (*G*^2^) and *p*-value associated with dropping each effect are reported in Table 6. In Model 3, the estimate of the *SD* of participant effect was 0.82 and for the accent effect, it was 0.89, showing that participants and accents account for a similar amount of variation in the model. That is, there is equivalent variability in terms of accuracy both across accents and across listeners.

**Table 6.** Summary of Likelihood Tests for the 2 × 2 Factorial Analysis: Accent.

Source	*df*	*G* ^2^	*p*
Listener nationality	1	1.62	.203
Accent nationality	1	4.02	.045
Listener nationality × accent nationality	1	8.05	.005

**Figure 3.**
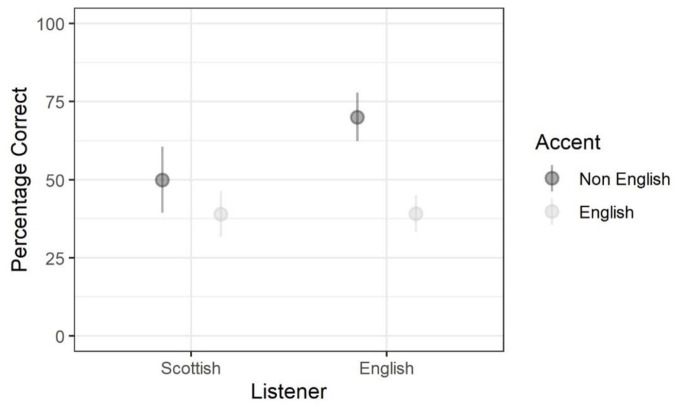
Accent identification accuracy.

There was no main effect of listener nationality; accuracy did not vary according to whether the listener was from England or Scotland. There was a main effect of accent nationality, with non-English accents identified more accurately than English accents. However, there was an interaction between listener nationality and accent nationality. Figure 3 aids interpretation of these results, showing the mean percentage accuracy in each condition of the factorial design. Based on visual inspection, English listeners were more accurate than Scottish listeners when identifying non-English accents but equally accurate when identifying English accents. As we show below, this effect can be understood better when we also consider how detailed these responses were.

The level data (Levels 1–5) were analyzed using multilevel ordered logistic regression in R using the ordinal package (Christensen, 2011). We tested for individual effects using the same method as the accuracy analysis. Three models were compared, this time with level as the dependent variable. The chi-square statistic (*G*^2^) and *p*-value associated with dropping each effect are reported in Table 7. In model 3, the estimate of the *SD* of participant effect was 0.69 and for the accent effect it was 0.75. As in the accuracy analysis, participants and accents account for a similar amount of variation in the model.

**Table 7.** Summary of Likelihood Tests for the 2 × 2 Factorial Analysis: Level of Region Specificity.

Source	*df*	*G* ^2^	*p*
Listener nationality	1	5.06	.025
Accent nationality	1	10.10	.001
Listener nationality × accent nationality	1	22.79	<.001

**Figure 4.**
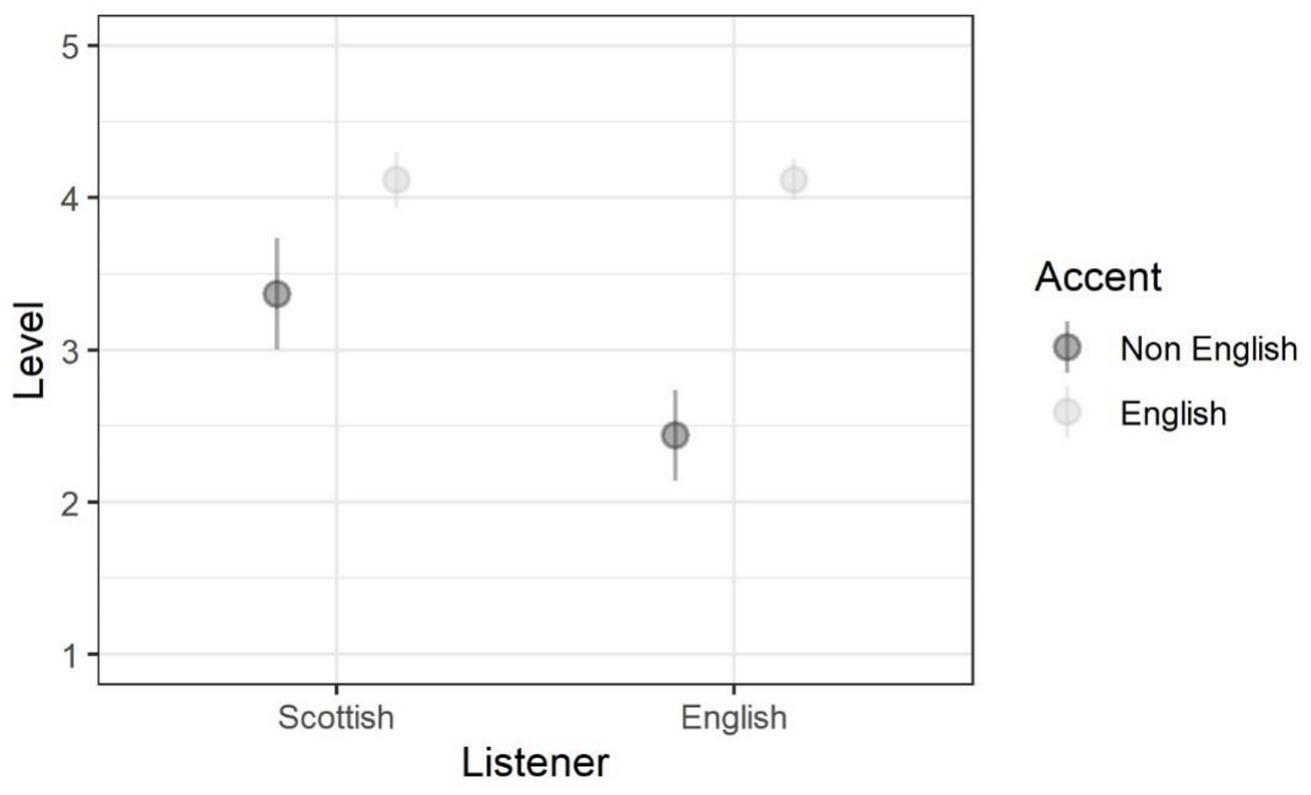
Mean response level of region specificity.

There was a main effect of listener nationality, with Scottish listeners being overall more specific than English listeners. There was also a main effect of speaker nationality, with non-English accents being identified at a less specific level than English accents. However, there was also an interaction. Figure 4 aids interpretation of these results, showing the mean level at which responses were provided in each condition of the factorial design. Based on visual inspection, Scottish listeners responded to non-English accents at a more specific level than English listeners and around the same level with English accents.

The relationship between level and accuracy was examined using the ordinal package in R (Christensen, 2011). Level was the dependent variable and accuracy was the predictor. Two models were compared, the first only included intercepts, and the second included accuracy as a predictor. Level predicted accuracy (*G*^2^ = 118.90, *p* < .001), with less specific responses more likely to be accurate. While 94.25% of responses at Level 1 were correct, only 31.48% of responses at Level 5 were correct.

The relationship between confidence and accuracy was examined using the same method as for the relationship between level and accuracy. Confidence predicted accuracy (*G*^2^ = 55.87, *p* < .001), with responses provided with higher levels of confidence more likely to be accurate.

All R-scripts and data can be accessed at: https://osf.io/ezmr3/?view_only=321a4d22b82745fda0fa54b617a99267.

In summary, the results of Experiment 2 are consistent with the results of Experiment 1, in that, accuracy was variable but tended to be low, and responses varied in terms of how specific they were. These data, using a different set of speakers, provides additional evidence for the conclusion that accent identification is difficult, but that some voices are more difficult to classify than others. As in Experiment 1, the results show that being less specific can help to mitigate the difficulty of accent identification, and people are aware of the difficulties associated with the task; confidence predicts accuracy. In addition, the results of Experiment 2 reveal that characteristics of the listener affect responses, both in terms of accuracy and how specific they are. Although English and Scottish listeners were just as accurate as each other when identifying English accents, and also provided similarly specific responses, there are marked differences when it comes to non-English accents. The accuracy analysis shows that English listeners were more accurate than Scottish listeners when identifying non-English accents, this can be accounted for by the fact that English listeners provided responses at a less specific level than Scottish listeners. Therefore, it appears that English speakers are more susceptible to the own-accent effect, which in this context is reflected by them being better at recognizing English accents at a specific level than non-English accents from further away. This might be explained by asymmetric exposure in the media; English listeners have less exposure to speakers with non-English accents than Scottish speakers do to English accents. We know that less familiar accents are detected at lower levels (Ikeno & Hansen, 2006) and people are better at recognizing accents that are closer to them (Clopper & Pisoni, 2004b) as well as familiar accents being easier to recognize (Arslan & Hansen, 1996; Braun et al., 2018; Hanani et al., 2013).

#### 4.4.2 Qualitative analysis

As stated in the methodology, we were interested to see which words and linguistic features the participants were using to help them make their judgments on where the speakers were from. We asked the participants to name any particular words in the reading tasks which assisted them, as well as allowing further qualitative comments about their way of speaking, other people they knew, and also in what way they had come across such varieties before. Previous studies have also been interested in such features. In the US study, Clopper and Pisoni (2004b) included a follow-up questionnaire which was completed after the perceptual categorization task. In this, listeners were asked to identify “properties that they had listened for in trying to categorize the talkers. The survey responses included such comments as specific sounds (‘o’ or ‘a’) or words (‘greasy’ or ‘wash’), as well as specific accents (New York or Boston, Southern ‘drawl’ or ‘twang’)” (Clopper & Pisoni, 2004b, p. 137). A study in the United Kingdom (McKenzie, 2015) also found that many participants described pronunciations of particular vowels and consonants to place speakers geographically. This shows that listeners do have some explicit knowledge and awareness of some of the properties that characterize regional accents.

Vowels are an important aspect of being able to recognize accents and in our task this was also the case. Many of the participants used vowels to help them decide where speakers were from. This included comments as simple as “vowels,” for example, in explaining how they knew a speaker was from Liverpool, Bolton, or Belfast (which were the three varieties with the highest comment of “vowels”). Particular words were also given to explain distinguishing features, such as “path” for London or “long vowel in path” for the RP speaker. In the case of the Birmingham speaker, one participant commented “path not paath.” In the case of the speaker from Carmarthen, participants commented that the “o” and “a” vowels were “long” and “extended.”

Certain consonantal features were also used to help decide where the voices came from. In the case of the Liverpool speaker, there were many comments that the “k” sounds in words like “book” sounded more like “x.” The consonant “t” was also used to distinguish accents, for some varieties, because they were “dropped” (e.g., London, Durham, and Birmingham) or because they were pronounced (in the RP speaker and Carmarthen). Rhoticity was a strong indicator of regionality, particularly for the parts of the country which pronounce /r/ following vowels in words, such as “air,” this applies to the Belfast, County Kerry, Glasgow, and Carmarthen speakers. It is interesting that such rhoticity was not mentioned for the speaker from Bristol, as this is also a typical feature of South-West England. In contrast, the RP speaker was said to produce /w/ rather than /r/ in post-vocalic environments. For the Birmingham and Nottingham speakers, a number of participants commented that word-final “g” was pronounced as “k” and “something as somethink.” H-dropping, l-vocalization (pronouncing “l” as “w” in words, such as “milk”), and th-fronting (pronouncing “three” as free’) were only mentioned for the Nottingham speaker.

There were large amounts of comments about overall speech patterns for individual speakers which were seen as typical for the region they were thought to come from. Many participants commented on the intonation patterns in Liverpool being very distinctive. This is one of the most typical features of Liverpool English and it seems it is recognizable to our participants (Wells, 1986, p. 373). Intonation was also frequently mentioned for both Irish speakers, with the Belfast voice said to be very distinctive, and “harsher” and “rougher” than the Republic of Ireland voice which was said to be “soft,” “softer than northern Irish,” “lyrical,” “lilting,” and “gentle and friendly.” The voice from Glasgow was said to be “clipped and clear,” “well spoken,” “soft,” and “attractive” and the Welsh voice “sing song,” “rhythmic,” “with a Welsh lilt” and “obvious.”

All voices from southern England, which includes London, Bristol, and RP (although not geographically southern, but shares many southern-like features) voices were said to be “well spoken,” “posh,” “polite,” “confident,” “subtle,” “well enunciated,” “Southern,” as well as having “little accent” or “few distinguishing features.” The voice from Birmingham was said to contain “some northern and some southern features” as well as being “brummy nasal.” The voice from Bolton was one of the few voices which referenced celebrities as participants stated it was like Peter Kay (who is from Bolton), John Cooper Clarke (who is from Salford near Manchester), and Ian MacMillan (who is from Yorkshire). Other comments included the soap opera “Coronation Street” (which is set in Manchester) and also that the speaker had “Yorkshire vowels.” The Nottingham voice was commented to be “too northern for Brummie,” “some northern and some southern features so hard to decide” but also to be “not well educated.”

We had asked our participants to let us know which particular words of the reading passage they had used to help them decide where the voices came from. The first noticeable difference is the amount of words given by participants to determine where speakers were from. The three accents with the most words given were Belfast, Carmarthen, and County Kerry (followed by Liverpool and Glasgow). These are the varieties that show most differences to Standard Southern British English (see, e.g., accent descriptions in Wells, 1986). The bottom three accents, so that, those with the least descriptors were London, Bristol, and the RP voice. These are all varieties of southern English and, so that, it seems that the participants found it harder to name particular features which made them recognizably different to more standard varieties. We can also look at the frequency with which certain words of the reading text were associated with particular accents. The difficulty we have with this is that we cannot be sure which particular aspects of the words on which the participants were focusing but comparing these results to the typical features discussed above helps us assess this. In future work, we would like to carry out experiments with real-time reactions to allow for a fuller analysis of which linguistic features are used to distinguish different varieties (e.g., Watson & Clark, 2015). For the three southern varieties (London, RP, and Bristol), “path” and “rainbow” were the two most frequent words given for these three varieties and no other accents had both these words in Top 2. We know particular words, such as “bath” are very strong indicators for differences between northern and southern varieties of English. It is interesting that these words are used to identify southern speakers only, and that the participants did not tend to use the “short a” produced by the other voices to differentiate them. For the Liverpool speaker, the words “look” and “act” were given most frequently as indicating this accent. Liverpool English is distinguished from other varieties of English in that voiceless stops (such as the sound “k”) lack full closure in word-final position, so that, sound more like “x” (see also Wells, 1986, p. 371). Both the Nottingham and Birmingham voices had the word “something” at the top of the list which ties in with the comments made about these speakers producing a sound which is more like “k” at the end. For the Nottingham voice, “horizon” was also one of the most common words named to identify this accent and this is a region where the “h” is frequently omitted, which was also mentioned by many of the participants. Words, such as “air” and “colour” featured highly for the Belfast, County Kerry, Glasgow, and Carmarthen voice. These are all rhotic areas, meaning that the /r/ sound is produced after vowels, which is no longer the case in many other areas of the United Kingdom.

**Figure 5.**
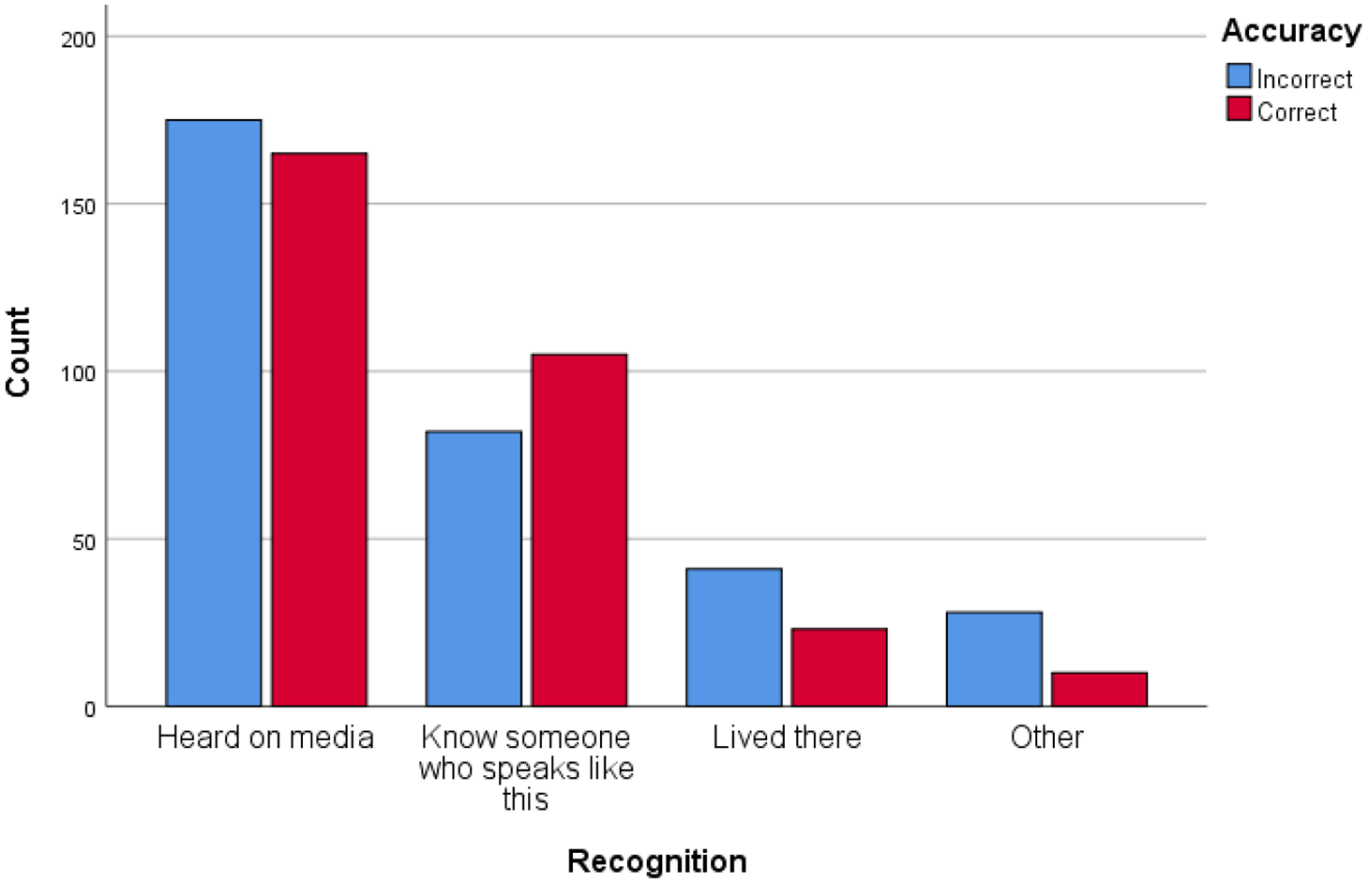
Accuracy versus recognition: all accents.

We had also asked participants where they had heard such an accent before (heard on media; know someone who speaks like this; lived there; other). Figure 5 shows that overall for all accents, only for the category “know someone that speaks like this” are speakers more likely to be accurate and/or more specific, but the differences are not great between any of the categories. This seems to suggest that having lived in an area does not help the participants in the accuracy of their recognition. This seems to contradict findings which suggest that residence can affect accuracy (Clopper & Pisoni, 2004a, 2004b). However, there is great variability between the different accents which must be considered.

**Figure 6.**
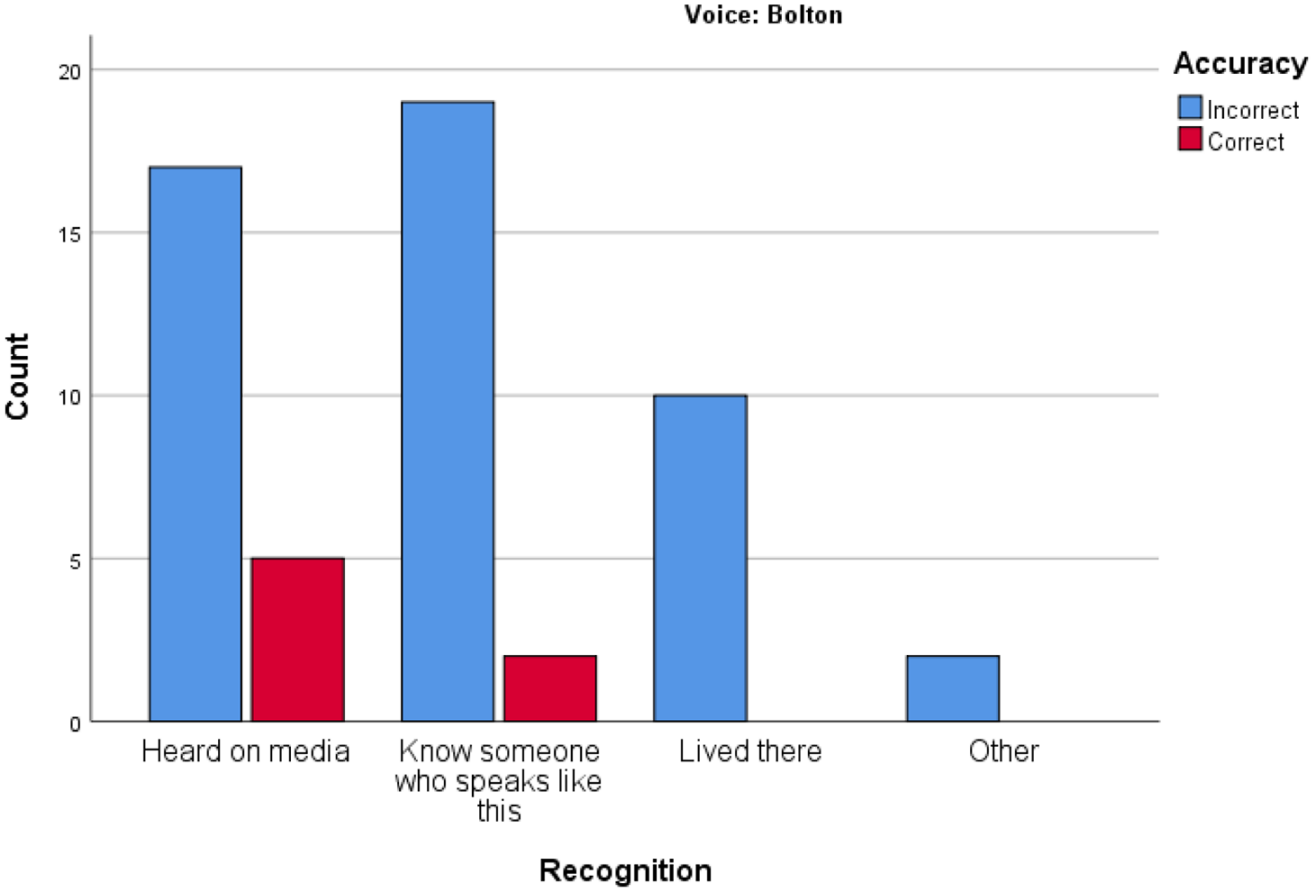
Accuracy versus recognition: Bolton.

**Figure 7.**
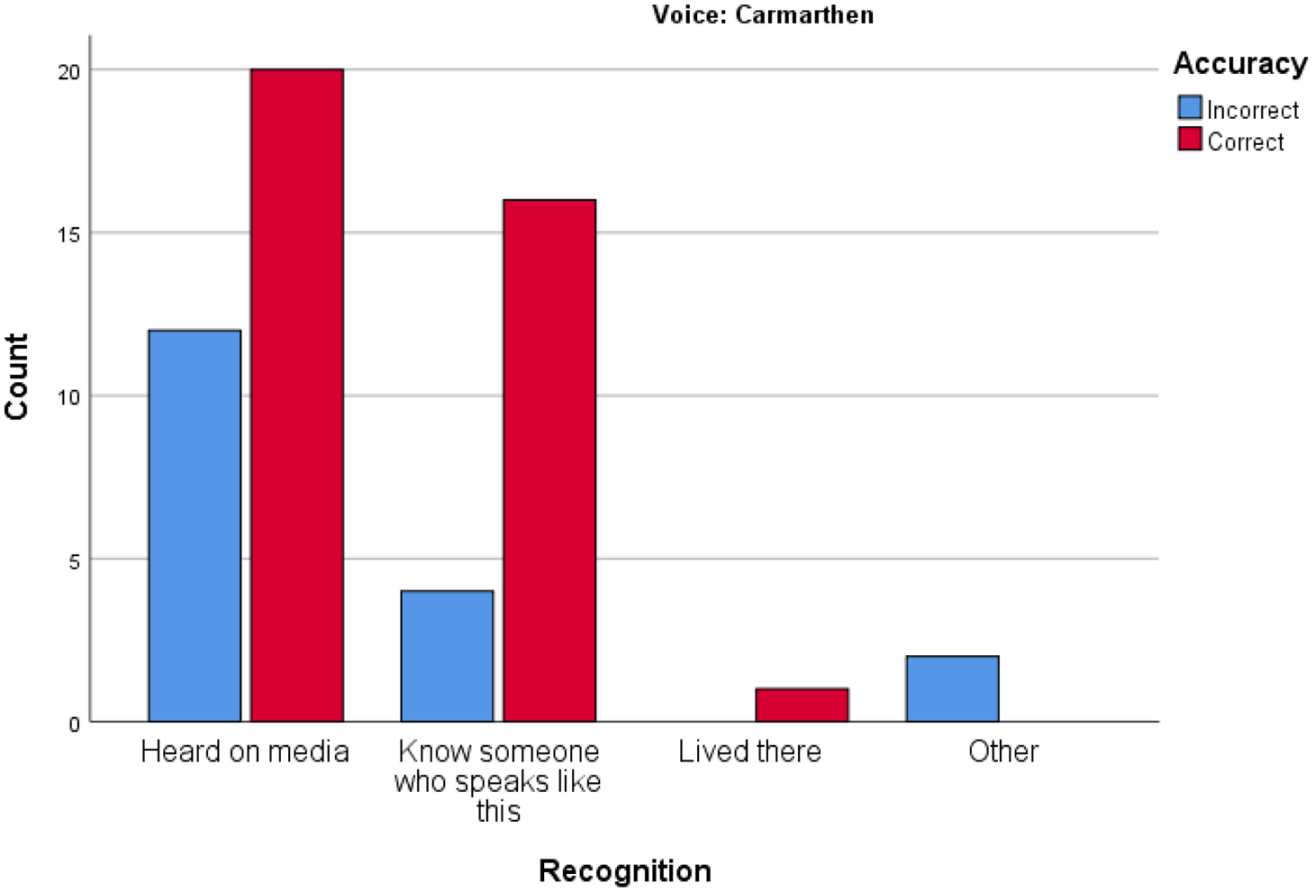
Accuracy versus recognition: Carmarthen.

Figures 6 and 7 relating to results for Bolton and Carmarthen, respectively, show the range of variability of accuracy when the participants say they recognize a voice because they know someone who speaks like this, or because they have lived somewhere. This is important from a forensic point of view. Familiarity of accents (due to having lived somewhere or having a friend with a particular accent) could be seen as indicators which predict accuracy and may be used by a witness to explain their knowledge. But it seems that these factors do not strongly predict accuracy, whereas confidence does seem a stronger predictor of accuracy.

## 5 Discussion and conclusion

This paper focused on accent, and listeners’ ability to recognize accents as they may be asked to do as part of their earwitness testimony through two different experiments. Accent is a very salient feature of language and we wanted to know more about how listeners respond to accents they hear. In the two experiments, we examined accuracy in accent recognition as well as the confidence in reporting accuracy. This has allowed us to build on what is already known about accent recognition to try to understand how this type of task can be applied in the real world, for example, when earwitnesses are asked about voices they may have heard to try to find the best way of gathering relevant and accurate evidence. Previous research has evidenced that people tend to be better at recognizing accents they are familiar with or which are closer to them to those they are less familiar with (Braun et al., 2018; Clopper & Pisoni, 2004b; Ikeno & Hansen, 2006), so that, we also wanted to examine whether previous exposure or familiarity had an effect on our participants. We also wanted to examine to what extent specificity of answers should be considered to ensure high accuracy while also providing police with useful information from earwitness statements.

Both experiments have shown that accuracy in recognizing voices from around the United Kingdom is generally low, but variable, supporting previous findings in Tompkinson and Watt (2018). Certain voices seem to elicit higher accuracy, for example, the voices from Ireland and Scotland are more often labeled correctly, but this is linked to the fact that these are given less detailed classification. This means that participants are more likely to use country descriptors, such as “Scotland” or “Ireland” for these voices, where they never use the descriptor “England.” For the voices from England, participants are more likely to provide more localized placements, such as particular smaller regions or cities. So, one of the novel findings of the present study is the demonstration through the comparison of different locations, that speakers are far more likely to give much broader descriptors for voices they are less familiar with. Confidence does seem to predict accuracy, with low confidence scores given to inaccurate voice placements and this pattern of results holds across a range of accents. This finding is in contrast to studies in the context of voice identification, which suggest that the relationship between confidence and unfamiliar voice identification accuracy can be weak (see Smith et al., 2020, p. 3). Although participants rarely have the meta-linguistic knowledge to accurately describe accents, it seems they are able to name particular features of the accent to justify their responses (frequently naming particular vowels or consonants that indicate how they have made their decision, as well as other linguistic features, such as intonation patterns). This could be taken into consideration when working with earwitness evidence, where witnesses could be asked to provide examples used when giving their descriptions or describing voices they have heard. When we compare the descriptions to the words they have chosen to justify their decisions, these are more extensive for non-familiar varieties. Especially for the English speakers, there seems to be an own-accent effect, where varieties more distant from standard, southern forms, are more difficult to place.

This research has shown us that although accent may be frequently reported by witnesses when describing a perpetrator’s voice, there are implications when using these in a forensic context. Currently available research suggests that earwitness evidence can be relatively error-prone, so that, cases should not be prosecuted solely on such evidence (Bull & Clifford, 1999, p. 203). This research suggests that participants are more accurate when less specific, which could suggest that police should encourage witnesses to be vague if they are not sure, or if they are not familiar with the accent. It could also suggest caution to police if witnesses are very specific. Where a prosecution case involves an assessment of whether a prosecution witness is accurate in their assessment of an accent, the jury should be warned about the increased risk which comes with greater specificity. However, we have also seen that confidence is related to accuracy, and in situations where participants are less confident, they are less likely to be accurate. We have seen that unfamiliar voices are much harder to place more specifically and that participants are more likely to give more general descriptions to voices they are not used to hearing. Having previous experience of accents does not always help participants place accents and there is great variability whether knowing someone with an accent or having lived in an area helps with perception. Although participants do not have the meta-linguistic ability to describe linguistic features of the voices they are hearing, they are able to pick out particular words and other linguistic features, such as intonation, which are indicative of particular varieties of English. Having both accuracy and specificity scores is a novel combination and allows us to have more detailed oversight into the levels of specificity participants’ use. These findings could be used as a basis for further research informing recommendations for police taking regional accent evidence from earwitnesses. This will help to ensure that such testimonies can be used in the most effective and accurate way. As well as the forensic applications of this research, it may also have wider implications in other fields, such as cross-linguistic communication, non-native accept perception and accent bias and discrimination. Making people aware that they may have certain preconceptions about certain varieties (see, for example, Coupland & Bishop, 2007, p. 74; Dixon et al., 2002, p. 162; Edwards, 1982, p. 20) and attribute certain attributes to particular speaker solely due to their accent (e.g., Dragojevic et al., 2021) may be able to help speakers overcome particular prejudices related to accent variation. Understanding that increased contact with other varieties can ease cognitive processes (see Ikeno & Hansen, 2006) can also be very valuable.

One of the limitations of these studies is the sample size and future research could gather larger amounts of participants to examine whether our findings are replicated across such sample sizes. It would also be interesting to examine in more detail the different levels of recognition which are given by participants to investigate how they vary and how this can be related more closely to accuracy. Future work could include examining in more detail individual listeners, to investigate whether some participants are better at recognizing accents generally as well as looking for more fine-grained patterns based on the regional accent of the participant taking part. Another important factor to consider is that we are asking participants to respond after hearing a voice and it would be interesting to be able to follow in real time the decisions participants make, in this way, we could examine which linguistic features they use to place an accent as it may be particular features they are unaware of and therefore not able to name when asked. Future work could also compare such free choice studies alongside studies where participants are given multiple choice options or using broader regions.

## Notes

1. Although the use of the term “lay listener” is not without controversy, this is the terminology used within the court system to distinguish witnesses who not fall within the specific category of “expert witnesses.” This categorization is adopted in the leading case of R v Flynn and St John [2008: EWCA Crim 970] and within the Crown Court compendium.
